# The human ortholog of the rodent testis-specific ABC transporter Abca17 is a ubiquitously expressed pseudogene (*ABCA17P*) and shares a common 5' end with *ABCA3*

**DOI:** 10.1186/1471-2199-7-28

**Published:** 2006-09-12

**Authors:** Armin P Piehler, Jürgen J Wenzel, Ole K Olstad, Kari Bente Foss Haug, Peter Kierulf, Wolfgang E Kaminski

**Affiliations:** 1 R&D Group, Department of Clinical Chemistry, Ulleval University Hospital, 0407 Oslo, Norway; 2 Department of Clinical Chemistry and Laboratory Medicine, Johannes Gutenberg University Hospital, 55101 Mainz, Germany; 3 Institute for Clinical Chemistry, University of Heidelberg, 68167 Mannheim, Germany

## Abstract

**Background:**

During the past years, we and others discovered a series of human ATP-binding cassette (ABC) transporters, now referred to as ABC A-subfamily transporters. Recently, a novel testis-specific ABC A transporter, Abca17, has been cloned in rodent. In this study, we report the identification and characterization of the human ortholog of rodent Abca17.

**Results:**

The novel human ABC A-transporter gene on chromosome 16p13.3 is ubiquitously expressed with highest expression in glandular tissues and the heart. The new ABC transporter gene exhibits striking nucleotide sequence homology with the recently cloned mouse (58%) and rat *Abca17 *(51%), respectively, and is located in the syntenic region of mouse *Abca17 *indicating that it represents the human ortholog of rodent *Abca17*. However, unlike in the mouse, the full-length ABCA17 transcript (4.3 kb) contains numerous mutations that preclude its translation into a *bona fide *ABC transporter protein strongly suggesting that the human *ABCA17 *gene is a transcribed pseudogene (*ABCA17P*). We identified numerous alternative ABCA17P splice variants which are transcribed from two distinct transcription initiation sites. Genomic analysis revealed that *ABCA17P *borders on another ABC A-subfamily transporter – the lung surfactant deficiency gene *ABCA3*. Surprisingly, we found that both genes overlap at their first exons and are transcribed from opposite strands. This genomic colocalization and the observation that the *ABCA17P *and *ABCA3 *genes share significant homologies in several exons (up to 98%) suggest that both genes have evolved by gene duplication.

**Conclusion:**

Our results demonstrate that *ABCA17P *and *ABCA3 *form a complex of overlapping genes in the human genome from which both non-coding and protein-coding ABC A-transporter RNAs are expressed. The fact that both genes overlap at their 5' ends suggests interdependencies in their regulation and may have important implications for the functional analysis of the disease gene *ABCA3*. Moreover, this is the first demonstration of the expression of a pseudogene and its parent gene from a common overlapping DNA region in the human genome.

## Background

ATP-binding cassette (ABC) transporters constitute a group of structurally related transmembrane proteins that translocate a vast variety of substrates across cellular membranes and thus perform essential biological roles in prokaryotic and eukaryotic cells [1]. The substrates include lipids, ions, sugars, peptides, vitamins, steroid hormones and xenobiotics such as anticancer drugs. Currently, the human ABC transporter family comprises 48 members [2,3]. Structurally, functional ABC transporters are characterized by two nucleotide binding folds (NBF) with conserved Walker A and B motifs, signature sequences, and two transmembrane domains (TMD), each typically composed of multiple transmembrane segments [1]. The energy required for the transmembrane transport of substrates is provided by hydrolysis of ATP at the two NBFs, whereas substrate binding is thought to be determined by the transmembrane and cytosolic domains. Functional ABC transporters require the presence of two NBFs and two transmembrane domains. This dual arrangement is accomplished either by a single polypeptide (a so-called full-size transporter) or by dimerization of two half-size transporters. Based on sequence similarities, the human ABC transporter family has been categorized into seven subfamilies, denoted ABC A-G. The human ABC A-subfamily presently comprises 12 full-size transporters [2,3] which share common structural features including a large extracellular loop between the first and second transmembrane helix and two conserved 80-amino acid segments adjacent to the NBFs. To date, four ABC A-subfamily genes have been causatively linked to monogenetic lipid trafficking disorders. These include familial high-density lipoprotein deficiency (caused by defective ABCA1), neonatal surfactant deficiency (ABCA3), several forms of retinal dystrophies (ABCR/ABCA4) and two types of hereditary keratinization disorders (ABCA12) [3].

Recently, a new member of the rodent ABC A-transporter subfamily, designated Abca17, has been identified which codes for a 1733 amino acid full-size transporter [4]. The function of Abca17 is presently unknown, however, its exclusive expression in spermatocytes suggests that this novel transporter may be implicated in lipid transport during spermatogenesis. In this study, we identified the human ortholog of Abca17. We report the surprising finding that the human equivalent of the rodent *Abca17 *gene is a transcribed, nonprocessd pseudogene (*ABCA17P*) which forms a unique complex of overlapping genes with the surfactant deficiency gene *ABCA3*.

## Results

### Bioinformatic prediction of human *ABCA17*

Homology searches based on the cDNA sequences of rat and mouse Abca17 and of human ABCA3, respectively, revealed the existence of a homologous gene in the human genome. The identified gene locus on human chromosome 16p13.3 exhibits 84.9%, 85.5% and 90.1% identity with the respective cDNA sequences of mouse/rat Abca17 and human ABCA3. The identification of several EST sequences with identities >99%, as retrieved by BLAT search (UCSC Genome Browser), provided an additional independent clue for the existence of a human homolog of the rodent *Abca17 *gene and it also strongly suggested that it was transcriptionally active. Moreover, homology analyses using the gene prediction program GNOMON resulted in the identification of a 3483 base pair (bp) cDNA sequence [GenBank:XM_372609] denoted "Homo sapiens similar to ATP-binding cassette, sub-family A member 3; ATP-binding cassette 3; ABC transporter 3 (LOC390669), mRNA". This sequence exhibits 100% identity with the retrieved genomic sequence which is composed of 21 exons. It contains an open reading frame encoding a putative 1160 amino acid (aa) polypeptide with two nucleotide binding folds and two transmembrane domains, each consisting of three membrane spanning segments and thus predicts a novel member of the ABC A-transporter subfamily.

### The human homolog of the rodent *Abca17 *gene is transcriptionally active but contains numerous mutations

To authenticate the existence of a transcribed human homolog of rodent Abca17, we performed RT-PCR using multiple sets of primers specific for the putative ABCA17 coding sequence. In fact, this approach confirmed that the human homolog of *Abca17 *is expressed and sequencing of overlapping PCR fragments revealed a full-length transcript of 4.2 kb size. The detailed cDNA sequence has been deposited with the NCBI GenBank Database [GenBank:DQ266102]. The 4.2 kb transcript is significantly shorter than that observed for mouse (5202 bp) and rat Abca17 (5322 bp), respectively, strongly suggesting that the human gene is truncated.

**Figure 1:**
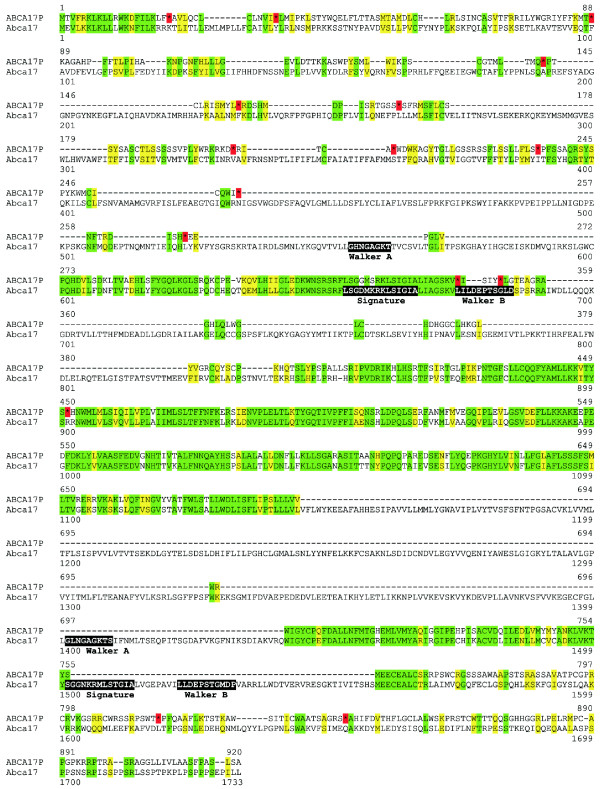
**Hypothetical amino acid sequence of human ABCA17P aligned with its murine ortholog Abca17**. The ABCA17P amino acid sequence shown is derived from cDNA segments with open reading frames. Gaps due to optimal alignment are represented by dashes and asterisks (red shaded) represent premature stop codons. Green and yellow shaded amino acids highlight sequence identities and similarities, respectively. Walker A and B motifs and signature sequences, integral components of the nucleotide binding domains of ABC transporters, are black shaded. The complete cDNA sequence of the ABCA17P full-length transcript has been deposited in the NCBI GenBank [GenBank:DQ266102].

Unexpectedly, detailed analysis of the human ABCA17 cDNA revealed the presence of numerous mutations including deletions, frameshifts and premature stop codons which preclude its translation into a functional ABC transporter protein product. Based on this, we refer to the human homolog of rodent *Abca17 *as "*ABCA17P*". An alignment of a hypothetical translation of the ABCA17P main transcript with the mouse Abca17 polypeptide sequence is shown in Figure 1 which details the topology of the ABCA17P mutations relative to its functional murine counterpart. The longest open reading frame is located in the center portion of the ABCA17P cDNA sequence and comprises 1092 bp. It predicts a hypothetical ABC transporter fragment of 361 amino acids size which contains three transmembrane spanning segments that corresponds to the N-terminal moiety of the second transmembrane domain of full-size ABC transporters (Figure 1).

As expected, the human ABCA17P nucleotide sequence displays highest identity with mouse (58%) and rat (51%) Abca17. These relatively low overall sequence homologies are not surprising because of the presence of several non-homologous gaps within the ABCA17P sequence. Consistent with this, more detailed segmental BLAT analysis revealed significantly higher homologies (78–90%) between the human *ABCA17P *gene and the corresponding exons of the mouse *Abca17 *gene (not shown). Moreover, interspecies comparison of the genomic microenvironments revealed that the human, mouse and rat genes are flanked by homologous genes indicating that the *ABCA17P *gene is indeed the human ortholog of the rodent *Abca17 *genes.

### Gene structure of human *ABCA17P *and identification of a novel mouse Abca17 exon 1

**Figure 2:**
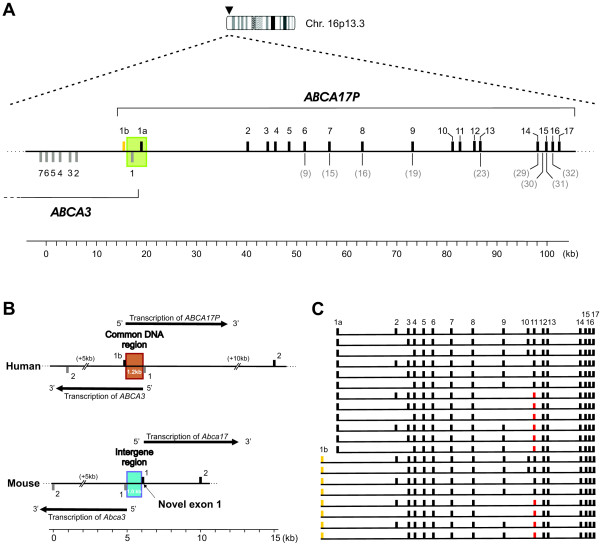
**(A) Structural organization of the human *ABCA17P/ABCA3 *gene locus on chromosome 16p13.3**. Both genes are organized in head-to-head orientation on opposite strands and overlap at their 5' ends. Exons are represented by black (*ABCA17P*) and gray boxes (*ABCA3*), respectively, and numbered in 5' to 3' order. Numbers in parentheses refer to *ABCA3 *exons that share >70% sequence homology with the *ABCA17P *exons indicated. The yellow box highlights the alternative exon 1b of the *ABCA17P *gene. The green box represents a common CpG island at the 5' end of both genes. A metric scale bar is shown. **(B) Comparison of the human and mouse *ABCA17 *– *ABCA3 *gene borders. **Shown are the first two exons of both genes and arrows indicate the respective directions of transcription. Note that the human *ABCA17P *and *ABCA3 *genes share a 1.2 kb overlap (brown box), whereas the murine *Abca17 *and *Abca3 *orthologs are separated by a 1.0 kb intergene region (blue box). An arrow identifies the novel exon 1 of the murine *Abca17 *gene. A metric scale bare is shown at the bottom. **(C) Synopsis of alternative ABCA17P transcripts identified in this study. **Shown are 20 ABCA17P transcript variants which are generated by alternative splicing of exons 2, 3, 9, 10 and a segment of exon 11 (red box), respectively. Boxes represent exons; the alternative first exon 1b is depicted by a yellow box.

The gene structure of the human *ABCA17P *gene was determined by BLAT analysis on the basis of the nucleotide sequence of the identified full-length transcript. The latter displayed 100% identity with a total of 17 adjacent genomic segments (= exons) which span an 88 kb region on chromosome 16p13.3 (Table 1, Figure 2A). Of note, systematic database searches and RT-PCR cloning experiments revealed transcripts that originate from an additional first exon (designated exon 1b, see below) positioned 1.8 kb upstream of the initially identified exon 1 (referred to as exon 1a) indicating that the human *ABCA17P *gene is in fact composed of 18 exons. The size of the human *ABCA17P *gene corresponds to that of other ABC A-subfamily members which ranges from 21 kb (*ABCA2*) to 449 kb (*ABCA13*). However, the fact that functional ABC A-transporters are routinely encoded by at least 38 exons the presence of only 17 exonic segments strongly suggests that *ABCA17P *is a truncated gene. Analysis of the exon/intron organization of *ABCA17P *demonstrated that exon sizes range from 85 to 1141 bp and introns vary in size between 0.2 and 21.8 kb (Table 1). All exon-intron boundaries showed the presence of the canonical dinucleotide GT-AG configuration which are diagnostic of splice junctions [5].

Surprisingly, close examination of the chromosomal microenvironment of the *ABCA17P *locus revealed that it borders on another ABC A-transporter gene, the lung surfactant deficiency gene *ABCA3*. Both genes are arranged in head-to-head orientation on opposite strands and overlap at their 5' ends (Figure 2A,B). Importantly, we found that the *ABCA17P *exon 1b is localized between exons 1 and 2 of the *ABCA3 *gene and the overlap region (distance between *ABCA17P *exon 1b and *ABCA3 *exon 1) spans 1.2kb. Thus, the human *ABCA17P/ABCA3 *locus represents a unique overlapping complex of a gene and its pseudogene from which both a protein-coding and a non-coding RNA are transcribed (Figure 2A,B).

**Figure 3:**
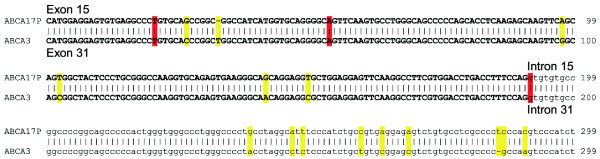
**Discrete regions of the *ABCA17P *gene show near perfect sequence identity with corresponding segments of the *ABCA3 *gene**. This is exemplified for *ABCA17P *exon 15 (+ partial intron 15) and *ABCA3 *exon 31 (+ partial intron 31), respectively. Bold capital letters represent exon and small letters intron sequences. Red shaded nucleotides indicate known mutation sites in the *ABCA3 *gene in individuals with neonatal surfactant deficiency. Yellow shaded letters denote non-identical nucleotides.

Beside the observation of overlapping 5' ends, we found significant overall homology between the human *ABCA17P *and *ABCA3 *genes (47%). Segmental sequence comparison revealed discrete regions of high sequence identity between both genes (Figure 3). For example, exons 6–9 and exons 13–16, respectively, of the *ABCA17P *gene exhibit sequence homologies ranging between 70% and 98% with distinct exons of the *ABCA3 *gene (Table 2, Figure 2A). Moreover, we observed that *ABCA3 *exon 30 is highly homologous to a sequence within intron 14 of the *ABCA17P *gene. These sequence homologies in exonic regions together with the chromosomal neighborhood of the *ABCA17P *and *ABCA3 *genes strongly suggest that both genes have originated by duplication of an ancestral gene.

Interestingly, cDNA sequence comparisons revealed that exon 2 of *ABCA17P *corresponds to exon 1 of the published rodent Abca17 gene suggesting the existence of an additional 5' exon in the rodent genome. Indeed, similarity searches in EST databases led to the identification of an EST sequence [GenBank:CB272331] which exhibits nearly 100% identity at its 3' end with the 5' portion of the published mouse Abca17 cDNA. RT-PCR and sequencing experiments confirmed the existence of the novel first exon in the mouse *Abca17 *gene (see NCBI [GenBank:DQ660313]). The novel exon 1 is localized 3.7 kb upstream of the previously published first exon and thus places the mouse *Abca17 *gene in closer proximity to its neighbor *Abca3 *with an intergenic region of only 1.0 kb (Figure 2B). Of note, the novel exon 1 displays only low homology (<45%) with *ABCA17P *exon 1a and 1b, respectively, indicating that the first exons of the mouse *Abca17 *gene and its human ortholog are structurally unrelated.

In the rat genome, the *Abca3 *and *Abca17 *genes are arranged in the same head-to-head orientation as in the mouse and human genomes. Both genes border on each other but do not overlap and each of the genes encodes a full-size ABC transporter. In addition, we also found evidence for tandem arrangement of the *Abca3 *and *Abca17 *genes in the dog and cow genomes (not shown).

To test whether or not the *ABCA17 *gene is intact in additional mammalian species, we assembled the putative ABCA17 cDNA sequences of dog, cow, chimpanzee and rhesus monkey, respectively, based on available genomic information and sequence identity with the mouse Abca17 cDNA. Using this approach, we identified a 5189 bp and a 5187 bp open reading frame in the dog and the cow which potentially encode ABCA17 full-size transporters in either species [see Additional file 1]. Moreover, EST database searches demonstrated the existence of several cow EST sequences with homologies >98% to the 5' and 3' ends of the predicted cDNA strongly suggesting that the cow *Abca17 *gene is transcribed. Conversely, analysis of the *ABCA17 *loci in the chimpanzee and the rhesus monkey genomes revealed numerous sequence alterations including preliminary stop codons, frameshifts, and splice site mutations in multiple exon candidates (not shown) consistent with the view that the *ABCA17 *genes of both of these primate species are indeed pseudogenes.

Intriguingly, during our cloning experiments we found evidence for multiple alternatively spliced transcripts of the human *ABCA17P *gene. Moreover, combined database searches and cloning experiments revealed the presence of an alternative transcription start site upstream of the one that we initially identified which initiates an alternative exon 1 ("exon 1b"). The sequence of the alternative exon 1b has been deposited in the NCBI GenBank under [GenBank:DQ660314]. Together, these findings predict that up to 20 RNA variants are transcribed from the human *ABCA17P *gene (Figure 2C). Our sequencing experiments using pooled cDNA derived from 50 individuals, also resulted in the identification of two single nucleotide polymorphisms (C2297T, G2326T) in the ABCA17P transcript.

### Tissue-specific expression of human *ABCA17P*

**Figure 4:**
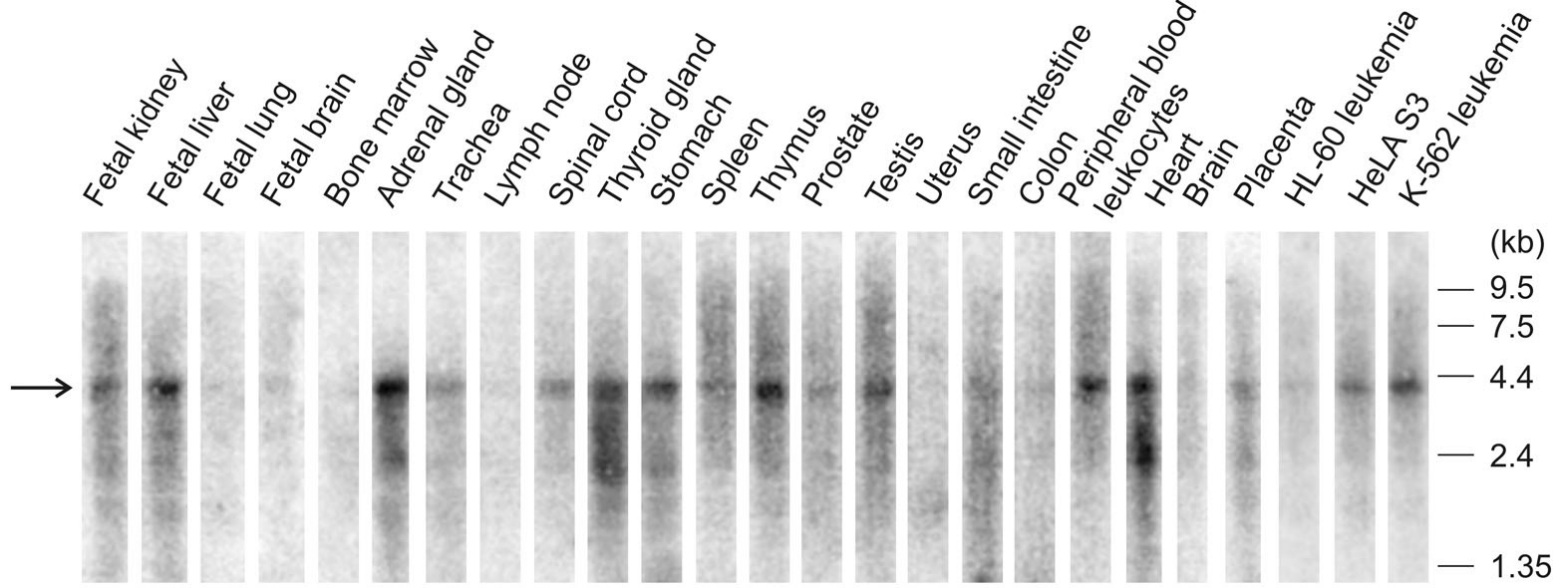
**Northern blot analysis of ABCA17P in various human tissues**. Northern blot demonstrating robust expression of the ABCA17P full-length transcript (arrow) in a variety of human tissues. A size marker is indicated for orientation.

The tissue-specific expression of human *ABCA17P *was characterized using Multiple Tissue Northern (MTN) Blots and the BD™ Multiple Tissue Expression Human Array 3 (MTE) containing poly-A^+ ^mRNA from various human tissues. Consistent with our RT-PCR cloning results, Northern blot analysis demonstrated the existence of an ABCA17P RNA of app. 4.3 kb size. This transcript was present in virtually all tissues examined (Figure 4). Highest expression was found in glandular tissue (salivary gland, adrenal gland and pancreas), heart (left ventricle and apex) and fetal tissues (kidney and liver) (Table 3). In contrast, ABCA17P RNA expression levels in testis and lung were rather low. The tissue-specific expression pattern of *ABCA17P *thus clearly differs from that of its genomic neighbor *ABCA3 *and also its rodent ortholog *Abca17 *which are almost exclusively expressed in the lung and the testis, respectively [4,6].

## Discussion

In the present study, we identified the human ortholog of the rodent ABC transporter Abca17. Unexpectedly, we found that this novel ABC A-transporter member is a ubiquitously transcribed pseudogene (*ABCA17P*) which structurally overlaps with the gene for the lung surfactant regulator ABCA3. The existence of a pseudogene homologous to *ABCA3 *(referred to as *ABCA3P1*) has recently been proposed in an *in silico *genomic survey [7]. The authors noted that *ABCA3P1 *consists of 7 exons and borders on the *ABCA3 *gene at a distance of 50 kb. Our results strongly suggest that *ABCA17P *and *ABCA3P1 *are identical, however, they indicate that *ABCA17P/ABCA3P1 *consists of a total of 17 exons and overlaps with the ABCA3 gene at their common 5' end.

Overlapping genes are often functionally or structurally related and genes transcribed from opposite strands of the same genomic locus may, depending on the exact structure of the locus, either regulate each other through antisense-based mechanisms, or may be coordinately regulated due to the common use of a bidirectional promoter [8]. An example for such a functional complex of overlapping genes is the *ABCG5/ABCG8 *locus. The *ABCG5 *and *ABCG8 *genes, which encode two highly homologous half-size ABC transporters, are juxtaposed on chromosome 2p21 and transcribed from a common bidirectional promoter which drives their coordinate expression. Our finding that the *ABCA17P *and *ABCA3 *genes overlap is thus not without precedence within the human ABC transporter gene family. However, the situation in the *ABCA17P/ABCA3 *locus clearly differs from that of the *ABCG5/ABCG8 *gene complex because *ABCA17P *and *ABCA3 *partially overlap in their transcribed regions and both genes exhibit significant differences in their tissue-specific expression patterns.

The immediate physical proximity of the human *ABCA17P *and *ABCA3 *genes is also observed in the mouse. Systematic analysis of the mouse *Abca17 *– *Abca3 *gene border resulted in the identification of a novel first exon of the *Abca17 *gene, which is located 3.7 kb upstream of the previously reported exon 1 [4]. Our findings narrow down the distance between the first exons of the *Abca17 *and *Abca3 *genes to a region of only 1 kb, however, we did not find evidence that both transcription units overlap. Consistent with this, the *ABCA17P *expression pattern differs considerably from that of its rodent ortholog *Abca17 *which is exclusively expressed in the testis [4]. It is thus likely that the overlap between the 5' ends of the *ABCA17P *and *ABCA3 *genes, which is absent from the mouse *Abca17/Abca3 *locus, constitutes the physical basis for the observed disparate tissue-specific expression of human *ABCA17P *and mouse *Abca17*.

The *ABCA17P/ABCA3 *locus is unique because it is the first demonstration in the human genome that a pseudogene and its progenitor gene overlap. To our knowledge, this is also the first example of a pseudogene/gene pair that is transcribed from a common 5' end. The significant homology between the *ABCA17P *gene and its neighboring progenitor *ABCA3 *and their colocalization in the genome strongly suggest that both genes arose by duplication of an ancestor gene. The fact that *Abca17 *and *Abca3 *are also neighbors in the rodent genome and the genomes of the dog and the cow, respectively, indicates that the gene duplication arose before the boreoeutherian radiation which has been dated to app. 85 Myr [9]. Moreover, our combined analyses of the genomes of the above species and those of the chimpanzee and the rhesus monkey suggest that the pseudogenization of the ABCA17 locus occurred after the divergence of rodents and primates.

Given the fact that mutations in the *ABCA3 *gene cause neonatal surfactant deficiency [10], our identification of the overlapping *ABCA17P/ABCA3 *locus confirms the observation that overlapping genes show a striking association with disease genes [11]. The distance between pseudogenes and their progenitor genes is inversely correlated with the likelihood for the transfer of mutations from a pseudogene to its functional gene by gene conversion [12]. For example, it has been shown that deleterious mutations from *ABCC6 *pseudogenes are transferred to their parent gene *ABCC6 *[13]. In contrast to the two known *ABCC6 *pseudogenes, which are located 1.3 Mb telomeric and 2.3 Mb centromeric of *ABCC6*, respectively, the distance between *ABCA17P *and its functional gene *ABCA3 *is zero. Thus, there is a high probability that *ABCA17P *serves as a repository for mutations in the *ABCA3 *gene. However, detailed sequence comparison between the known mutated exons in *ABCA3 *and their corresponding exons in *ABCA17P *revealed that all nucleotide positions in the *ABCA17P *gene that correspond to mutation sites in *ABCA3 *represent wildtype sequences. Moreover, *in silico *searches for gene conversion events in the *ABCA17P *exons 6–9 and 13–16, which share highest homology with *ABCA3 *exons, utilizing the program GENECONV did not yield statistically significant results (not shown). Together, these findings do not support the view that sequences of *ABCA17P *may have contributed mutations to *ABCA3*.

Importantly, we also found that *ABCA17P *and *ABCA3 *genes share discrete regions of high homology. In particular, exons 8 and 15 of the *ABCA17P *gene exhibit up to 98% sequence identity with the *ABCA3 *exons 16 and 31, respectively. The existence of these highly homologous DNA segments in both genes has immediate practical relevance because accidental amplification of *ABCA17P *sequences may provide erroneous results upon mutation analysis of the *ABCA3 *gene. Because *ABCA17P *is transcriptionally active in a broad spectrum of human tissues including the lung, the major production site of ABCA3, it also needs to be taken into account that ABCA17P transcripts may potentially interfere with ABCA3 mRNA expression analyses due to cross-hybridization of ABCA3 specific probes or RT-PCR primers with homologous pseudogene sequences.

Although recent computational approaches estimate that the number of pseudogenes in the human genome ranges between 14,000 and 19,000 [12,14,15], only little is known about their biological significance. In particular, there is a great paucity of information on transcriptionally active pseudogenes. Considering that the most comprehensive study thus far focussing on transcribed pseudogenes suggests that up to 20% of the pseudogenes on chromosome 22 may indeed be transcriptionally active [16], it is tempting to speculate that transcribed pseudogenes may serve defined biological functions rather than represent mere genomic fossils. This view is convincingly supported by a recent landmark paper that provided evidence for a specific function of a transcribed pseudogene by demonstrating that the expressed pseudogene *makorin1-p1 *regulates the stability of the mRNA of its progenitor gene *makorin1 *[17]. Another mechanism of specific pseudogene-gene interference has been identified for the antisense pseudogene *anti-NOS *whose transcript forms stable RNA-RNA complexes with the mRNA of *nNOS *and thus inhibits protein expression of its parent gene [18].

At this point, the exact function of the *ABCA17P *gene is unclear. However, *ABCA17P *stands out among the known transcribed pseudogenes in that it shares its 5' end with its functional counterpart. Due to this unique genomic architecture the putative *ABCA17P *promoter is part of the *ABCA3 *gene and vice versa. Moreover, the primary transcripts derived from both genes have a 1.2 kb overlap. It is thus conceivable that *ABCA17P *and *ABCA3 *may mutually impact their promoter activities and the elongation rates of their primary transcripts due to steric interference of their transcription machineries. Such a scenario is supported by the largely complementary expression patterns of both genes in human tissues. More work is required to determine whether and to which degree *ABCA17P *is involved in the control of the disease gene *ABCA3*.

## Conclusion

In this study, we identified the primary structure of the human ortholog of the recently cloned murine ABC transporter Abca17 and demonstrate that, unlike its rodent counterpart, it is a ubiquitously expressed pseudogene. Surprisingly, we found that *ABCA17P *overlaps with the gene for the lung surfactant regulator ABCA3 and sequence homology analyses strongly suggest that *ABCA3 *is the progenitor gene of *ABCA17P*. To our knowledge, this is the first example of the co-expression of a pseudogene and its progenitor gene from a common overlapping DNA region in the human genome. These unique genomic features suggest potential interdependencies between the regulation of *ABCA17P *and the disease gene *ABCA3*.

## Methods

### Identification of the human ABCA17P gene and its mammalian orthologs

The human *ABCA17P *gene was identified by systematic similarity searches using the BLAT [19,20] and the ParAlign [21,22] software, respectively. The query sequences included the Abca17 cDNAs from rat [GenBank:AB196699] and mouse [GenBank:AB112584] and the human ABCA3 cDNA [GenBank:NM_001089]. The genomic sequence of the human *ABCA17P *locus was narrowed down by the adjacent genes *ABCA3 *and *CCNF *[GenBank:NM_001761] and extracted from the UCSC Genome Browser (assembly May 2004, NCBI Build 35). For identification of available EST sequences specific for human *ABCA17P*, the genomic sequence of the *ABCA17P *locus was analyzed utilizing ParAlign and the UCSC Genome Browser. The gene structure of the human *ABCA17P *gene and its genomic microenvironment was determined on the basis of the cloned human ABCA17P cDNA sequence using the BLAT algorithm. Potential CpG islands were identified in a genomic region extending from 2 kb upstream of the transcription start site to the first intron of *ABCA17P *using the CpG Island Searcher [23,24]. Detailed homology comparisons and multiple sequence alignments were conducted utilizing the GAP (WWW2 HUSAR GCG software, DKFZ, Heidelberg) [25] and EMMA algorithms (EMBOSS) [26,27], respectively. The programs SIXPACK and TMAP (EMBOSS) were used to establish open reading frames within the ABCA17P transcripts and identify potential transmembrane domains. The *ABCA17 *genes of cow, dog, chimpanzee and rhesus monkey, respectively, and their respective putative cDNAs were identified on the basis of available species-specific genomic sequence information and sequence homologies with the known human and rodent ABCA17 cDNAs. For this the conservation track in the UCSC Genome Browser was used. For statistical prediction of gene conversion events in the human *ABCA17P *and *ABCA3 *genes the software GENECONV [28] was utilized.

### Assessment of human ABCA17P full-length cDNA sequence

Total RNA from human testis (pooled from 50 individuals) was purchased from Clontech and aliquots of 1 μg were reverse-transcribed in the presence of oligo-dT primers in a 20 μl RT reaction using the Omniscript RT Kit (Qiagen). cDNA was amplified using the Taq PCR Core Kit (Qiagen). The cycling conditions were 94°C 30s, 54–60°C 30 s and 72°C 30s-3min followed by a final extension step at 72°C for 10 min. ABCA17P-specific cDNA segments were amplified utilizing sets of primers with highest homology to the coding region of rat and mouse Abca17, respectively. Missing interspersed segments were amplified using primers specific for the identified fragments and published EST sequences. Amplification primers which resulted in the identification of alternatively spliced RNA variants were TCACGCACTGTCTTTCCTTG/CCAGTACAATGGAACTGATGATG (ABCA17PΔ-85, ABCA17PΔ-213), GCCCTGAAGTCAAGCAGGT/CCAATGGGACATTTTCTATGC (ABCA17PΔ-162) and TGAGGTCCCATAGCAGAGTG (ABCA17PΔ-387, ABCA17PΔ-486), respectively. The alternative transcription start was identified using the primers CGTGCTACCCTCTCCTGTTC/TGCACCAGCCTTCTATGTCA. All PCR amplification products were separated and analyzed on GelStar^® ^(Cambrex) stained 0.8 – 1.5% agarose gels. Bands of interest were excised and the DNA was extracted using the QIAquick^® ^Gel Extraction Kit (Qiagen). PCR runs resulting in a single band were purified using Microcon^® ^YM-30 columns (Millipore) for subsequent sequence analysis. All amplification reactions were sequenced at least in duplicate.

### DNA sequencing

PCR products of interest were purified and desalted using Microcon^® ^YM-30 columns (Millipore). Purified amplification products were sequenced according to standard protocols as published previously [29]. Sequence data were analyzed using the program FinchTV v.1.3.1 (Geospiza Inc.) and the UCSC Genome Browser.

### RNA expression analysis of ABCA17P in human tissues

To authenticate the length of the ABCA17P primary transcript, Northern blot was performed utilizing commercial multiple tissue blots (Clontech) which contained at least 1 μg poly-A^+ ^mRNA from 22 different human adult tissues, four different human fetal tissues and 8 different human cancer cell lines. The tissue specific expression of *ABCA17P *was characterized using the BD™ MTE Multiple Tissue Expression Human Array 3 (Clontech). The array is normalized to the mRNA expression levels of eight different housekeeping genes and thus allows for semiquantitative poly-A^+ ^RNA expression profiling in >70 distinct human tissues. A 0.4kb ABCA17P specific probe was generated by RT-PCR using primers that were derived from a region of minimal homology between ABCA17P and other ABC A-transporter nucleotide sequences. The primers used were TGCGCTTCAGTTACTTTCAGA and CCAGTACAATGGAACTGATGATG. For signal detection, the probe was radiolabeled with Ready-To-Go™ DNA Labeling Beads and [α-^32^P]-dCTP (Amersham Biosciences) and hybridization and washing steps were performed according to the manufacturer's protocol. Hybridization signals were visualized and quantitated in an electronic autoradiography system (Packard InstantImager). Individual activity signals were translated into a linear integer scale ranging from 0 to 4 (0, no expression; 4 maximum expression) as previously described [29].

## Authors' contributions

KBFH, PK, WEK supervised the project. APP, PK, WEK designed the study. APP, JJW performed bioinformatic analyses and conducted cloning experiments. APP, OKO performed RNA expression profiling experiments. APP, KBFH, JJW, OKO, WEK analyzed the data. APP, JJW, WEK prepared the manuscript. All authors read and approved of the final manuscript.

## Supplementary Material

Additional File 1 Comparison of the Abca17 polypeptide sequences of mouse, rat, dog and cow. This figure shows an alignment of the Abca17 polypeptide sequences of mouse, rat dog and cow. The amino acid sequences of mouse and rat Abca17 were obtained from published GenBank entries. The putative Abca17 polypeptide sequences of the dog and the cow, respectively, were generated on the basis of nucleotide sequence homology searches in the respective genomes. Click here for file

## Figures and Tables

**Table 1:** Exon-intron boundaries of the human *ABCA17P* gene.

Exon	Exon size (bp)	5' splice site	3' splice site	Intron size (kb)
1b	n.d.		CAGAATGGTGgtaagtcact	21.8
1a	452		CCCCAGCCGGgtaggagtca	19.7
2	85	tcctgactagTGATGAGCCC	CTTAAAGTTGgtatggttat	4.5
3	128	ctctctgtagTTTTAGGCTG	GCCACTTGCGgtaagaacat	1.2
4	163	ttcttcatagGTTAAGTATC	GGAAGTCCTGgtgagaaagc	3.2
5	260	tttctcatagGATACAACAA	AAGACTGAAGgtaaccccag	3.4
6	117	ctttctctagGATTACCTGT	CTGCACAGAGgtacatatct	5.4
7	154	ttggttctagGTCTTATTCC	CTATGGTCAGgtgagtcgat	6.5
8	153	gtcgttgcagCTGAAGGGCC	AGGCTCCAAGgtgcggcggt	9.6
9	99	ttccttccagGTTTGAATCT	TCTTCATAAGgtaagcacgt	8.1
10	145	atgttggcagATGCCAGTAC	AAACACTGGGgtaaataact	2.4
11	307	tttattgtagTTCAGTCTTC	GAGGTCCTAGgtaagtatgt	3.3
12	324	tcccttctagGTTCTGTAGA	TCCTGTATCAgtatgtgtga	0.2
13	205	tcttttacagGGAACCTAAA	GCTGCTGGTGgtgagtgctg	11.8
14	188	tctgacccagGTATGGCGGT	AGACCTACAGgtgagatctg	1.8
15	190	ctctccccagCATGGAGGAG	ACCTTTCCAGgtgtgtgccg	0.2
16	110	cttcgtgcagGCAGCGTTCT	ACTTTCTGGGgtaactctgt	0.5
17	1141	ctcccctcagGTGTTTGGCA		

1a, b: alternative first exons; n.d. not determined

**Table 2:** *ABCA17P *and *ABCA3 *exons sharing significant homologies

*ABCA17P*	*ABCA3*	DNA sequence homology
Exon 2	Exon 4	61.3
Exon 3	Exon 6	53.1
Exon 4	Exon 7	60.1
Exon 5	Exon 8	63.1
**Exon 6**	**Exon 9**	**70.1**
**Exon 7**	**Exon 15**	**75.3**
**Exon 8**	**Exon 16**	**98.0**
**Exon 9**	**Exon 19**	**75.8**
Exon 10	Exon 20	43.5
Exon 11	Exon 21	55.9
Exon 12	Exon 22	60.9
**Exon 13**	**Exon 23**	**75.6**
**Exon 14**	**Exon 29**	**73.4**
**Exon 15**	**Exon 31**	**97.4**
**Exon 16**	**Exon 32**	**90.5**
Exon 17	Exon 33	42.9
		
Intron 14	Exon 30	88.9

*ABCA17P *and *ABCA3 *exons displaying sequence identities >70% are bolded. Note the high homology between *ABCA17P *intron 14 and *ABCA3 *exon 30.

**Table 3:** Ubiquitous expression of ABCA17P RNA in human tissues

Tissue	RNA expression	Tissue	RNA expression
Whole brain	•	Lung	•
Cerebral cortex	•••	Placenta	•
Frontal lobe	••	Bladder	••
Parietal lobe	••	Uterus	••
Occipital lobe	•	Prostate	•
Temporal lobe	•	Testis	•
Postcentral gyrus	•	Ovary	•
of cerebral cortex		Liver	•
Pons	•	Pancreas	•••
Cerebellum left	•	Adrenal gland	•••
Cerebellum right	••	Thyroid gland	••
Corpus callosum	•••	Salivary gland	••••
Amygdala	••	Mammary gland	•
Caudate nucleus	•	Esophagus	•
Hippocampus	•	Stomach	•
Medulla	•	Duodenum	••
oblongata		Jejunum	•••
Putamen	•	Ileum	••
Substantia nigra	•	Ileocecum	•
Accumbens	••	Appendix	-
nucleus		Colon, ascending	•
Thalamus	••	Colon, transverse	•
Pituary gland	•	Colon, descending	•
Spinal cord	•	Rectum	•
Heart	••	Fetal brain	•
Aorta	•	Fetal heart	••
Artrium left	••	Fetal kidney	•••
Artrium right	••	Fetal liver	•••
Ventricle left	••••	Fetal spleen	•
Ventricle right	••	Fetal thymus	•
Interventricular	••	Fetal lung	•
septum		Promyelocytic leukaemia,	•
Apex of the heart	••••	HL-60	
Kidney	•	Hela S3	•
Skeletal muscle	•	Chronic myelogenous	••
Spleen	•	leukaemia, K-562	
Thymus	••	Lymphoblastic leukaemia,	••
Peripheral blood	•	MOLT-4	
leukocyte		Burkitt's lymphoma, Raji	•
Lymph node	•	Burkitt's lymphoma, Daudi	•
Trachea	•		

ABCA17P RNA expression for individual tissues was determined by dot blot and quantitated using electronic autoradiography. The relative signal intensities were translated into a linear scale (0–4). -, not expressed.
