# Supplementary material for: The human ortholog of the rodent testis-specific ABC transporter Abca17 is a ubiquitously expressed pseudogene (ABCA17P) and shares a common 5' end with ABCA3
Source: BMC Mol Biol. 2006 Sep 12;7:28. doi: 10.1186/1471-2199-7-28 (PMC1579226; doi:10.1186/1471-2199-7-28)
Supplement: Additional File 1 — Comparison of the Abca17 polypeptide sequences of mouse, rat, dog and cow. This figure shows an alignment of the Abca17 polypeptide sequences of mouse, rat dog and cow. The amino acid sequences of mouse and rat Abca17 were obtained from published GenBank entries. The putative Abca17 polypeptide sequences of the dog and the cow, respectively, were generated on the basis of nucleotide sequence homology searches in the respective genomes. [file 1471-2199-7-28-S1.pdf]

Additional file 1. Comparison of the Abca17 polypeptide sequences of mouse, rat, dog and cow.

```
   1                                                                                                  100
Mm MEVLKKLKLLLWKNFILKRRKTLITLLEMLMPLLFCAIVLYLRLNSMPRKKSSTNYPAVDVSLLPVCFYNYPLKSKFQLAYIPSKSETLKAVTEVVEQTF
Rn MAPFKKLKLLLWKNFVLKKRKTLVTVLETLMPVLFSAIVLYLRLNSMPRNKANTNYPAVDVSLLPVYFHNYPLKSKFQLVYIPSKSETLKAVTEVVEQTF
Cf MTLGRNLKLLLWKNFILKKRKPLVTMLEVLAPLLFSMIIMYLRLNSVPRERPPMSYLALNVSLLPDFL.RLAAQSTYQLVYVPSQSDTLKAVTEAVAKAF
Bt MAMFRNLKLLLWKNFILKKRKTLVTVLEILMPLLFCAVIIYLRFGSLPRKRPPVDYKVIDITSLPEFFNQFPLKNKFQLVYIPSKSETLKNITEMVKDFF

   101                                                                                                200
Mm AVDFEVLGFPSVPLFEDYIIKDPKSFYILVGIIFHHDFNSSNEPLPLVVKYDLRFSYVQRNFVSPPRHLFFQEEIEGWCTAFLYPPNLSQAPREFSYADG
Rn AVDFEVLGFPSVSLFENYIIKDPKSFYVLVGIVFHHDFNSSNEPLPLVVKYDLRFSYVQRNSISPPRHLFFQEDIEGWCTAFLYPPNLSQAPREFSYADG
Cf EVEFEVLGYPSVPLFENYVIQDPRAFYVLAGIVLDHSFNDRQQPLPLEIKYRLRFSCIQRNSLT.SKLTSYYDTLEGWGTSLLYPRNPSQAPRAFGQIDG
Bt TVEFEVLGYPSVPVFEKYIIQDPKAFYVLTGIVFDHNFNDNTEPLPLEVKYYLRFSFFQRNYLS.LRLILQQDDMQGWLTSFLYPPNLSQEPRDYGVSEG

   201                                                                                                300
Mm GNPGYNKEGFLAIQHAVDKAIMRHHAPKAALNMFKDLHVLVQRFPFGPHIQDPFLVILQNEFPLLLMLSFICVELIITNSVLSEKERKQKEYMSMMGVES
Rn GHPGYNKEGFLAIQHAVDKAIMLHHAPKAALDMFKNLQVSVQRFPSGSHIQDPFLVILQNEFPLLLMLSFICVELIITNSILLEKERKQKEYMYLMGLEN
Cf GLPGYNQEGFLAIQHALDKAIMQHHAHNATTRMFEDLTVLVKRFPHGAHIQDKFLLILQNEFPLLLMLSFICIELIIINSIALEKERKMKEYMCMMGVNN
Bt GAPGYYQEGFLAIQHATDRAIMWHHAPTETTGLFQSLSVLLKRFPHGAYVRDIFFLVLQNEFPVFLMLSFICIELITINSIVLEKERKLKEYMCMMGLHS

   301                                                                                                400
Mm WLHWVAWFITFFISVSITVSVMTVLFCTKINRVAVFRNSNPTLIFIFLMCFAIATIFFAFMMSTFFQRAHVGTVIGGTVFFFTYLPYMYITFSYHQRTYT
Rn WLHWVAWFITFFLSALVTVSGMTVLFCTKMNGVAVFRNSNPTLIFIFLMCFAIATIFFAFMMSTFFQRAHVGTVIGGIVFFFTYLPYMYITFSYHQRTYS
Cf WQHWAAWFIMFFISTLIVISFMTILFCIEVHGVVVFRNSDPSLIFVFLLCFATATILFAFMISTFFQKAHVATAAGGIIFFFTYLPYLYLTFSYPQRTYF
Bt WQHWVAWFIVFFISASIVVSFMTLLFCIEFDESAVFGNSDPSLIFVFLMCFAIATIFFAFMISTFFQKAHVATASGGIIFFFTYLPYLYIAFTYSQRSSF

   401                                                                                                500
Mm QKILSCLFSNVAMAMGVRFISLFEAEGTGIQWRNIGSVWGDFSFAQVLGMLLLDSFLYCLIAFLVESLFPRKFGIPKSWYIFAKKPVP......EIPPLL
Rn QKILSCLFSNVAMAMGVRFISLFEAEGTGIQWRNMGSVWGDFSFTQVLVMLLLDSFLYCLVAFLVESLFPRKIGMPKSWYIFAKCPLWRKKSFPVIPPLL
Cf QKVAFCLLSNIAMALGVRLISSFEIRGTGMRWKHVGGLSGEFNFSHVLVMLVLDSVLYGLVAWYVEAVLPGEYGIPKPWYFFVMPSHWQGRSSSLTRSVL
Bt QKIAFCLLSNVAMALGVRLISTFEARGTGVQWRNMGHIGGEFNFTQVLLMLLLDSVLYGLVAWYVEAIFPGEYGIPKPWYFFLTPSYWWREPTSLRNPVE

   501                                                                                                600
Mm NIGDPEKPSKGNFMQDEPTNQMNTIEIQHLYKVFYSGRSKRTAIRDLSMNLYKGQVTVLLGHNGAGKTTVCSVLTGLITPSKGHAYIHGCEISKDMVQIR
Rn VIGDPEKTSKGDFLQDEPAGHINAIEIQHLYKVFYTGRSKCIAVKDLSMNLYKGQITVLLGHNGAGKTTVCSVLTGLIPPSKGHAYIHGCEISKDMVRIR
Cf DVGDPDKASESKFIQEEPTDLAKGIEIRQLYKVYQTGKSKHVAVKGLTMNLYQGQITVLLGHNGAGKTTMCCMLTGVLAPSSGWVYINGYDISCNVAQIR
Bt DLEDPQQALGNKFIQDEPTNLIKGIEIQHLYKVFYKGRDEHVAVKDLTVNLYQGQITVLLGHNGAGKTTTCNILTGLTTPSRGQVFINGYELSKDMLQIR
                                                              Walker A

   601                                                                                                700
Mm KSLGWCPQHDILFDNFTVTDHLYFYGQLKGLSPQDCHEQTQEMLHLLGLKDKWNSRSRFLSGDMKRKLSIGIALIAGSKVLILDEPTSGLDSPSRRAIWD
Rn KNVGWCPQHDILFDNFTVTDHLYFYGQLKGLSHQDCHEKIEEMLHTLGLEDKRNSRSKFLSGGMKRKLAIGIALIAGSKVLILDEPTSGMDSSSRRAIWD
Cf KSMGWCPQHDILFDNFTVAEHLYFYARLKGLSCHKCPEEVQRMLHVLGLEDKRDALSKFLSGGTKRKLSIGIALIAGSKVLMLDEPTSGMDAISRRAIWD
Bt KSMGWCPQHDILYDNLTVAEHLYFYAQLKGLPRQKCPEEVKRMLHVLGLEEKRDSRSRFLSGGMRRKLSIGIALIAGSKVLMLDEPTSGVDAISRRAIWD
                                                                       Signature          Walker B

   701                                                                                                800
Mm LLQQQKGDRTVLLTTHFMDEADLLGDRIAILAKGELQCCGSPSFLKQKYGAGYYMTIIKTPLCDTSKLSEVIYHHIPNAVLESNIGEEMIVTLPKKTIHR
Rn LLQQQKGDRTVLLTTHFMDEADLLGDRIAILAKGELQCCGTPSFLKQKYGAGYYMTIIKTPLCDTEKLAKVIYHHIPNAILESRIGEEMIFTLPKKAMPR
Cf LLQQHKSDRTVLLTTHFMDEADLLGDRVAIMAKGELQCCGSSLFLKQKYGAGYYMTLVKKPHCSTEKISHLIYQHIPNAVLQSSTGEELTFILPKKNIHR
Bt LLQQHKSDRTILLTTHFMDEADLLGDRIAIMAKGELQCCGSSLFLKEKYGAGYYITLVRKPHCDTEKISLLVYQHIPNAVFQSSIGEELTFILPKESAHS

   801                                                                                                900
Mm FEALFNDLELRQTELGISTFATSVTTMEEVFIRVCKLADPSTNVLTEKRHSLHPLPRHHRVPVDRIKCLHSGTFPVSTEQPMRLNTGFCLLCQQFYAMLL
Rn FEALFADLEQRQTELGISTFGASVTTMEEVFIRVCKLADPSTNVLTEKRPSLRHLPRNHRVPVDRIKCLHSRIFSLSSDQPIRLNTGFSLLCQQFYAMLL
Cf FARLFTELERRQVELGVASFGVAVTTMEEVFVRVNKLSDTSTDIQALKTSSMQSQ...LRIPISRIKRFHSRIFSLRSGLPIHMNTGLSLICQQFYAMLL
Bt FEALFTDLELRQEELGIASFGASVTTMEEIFIRVNKLMDSDIDLQIIKLPSFHSHPLVSRRVPVNRIRRLHSRIFSIPSGLPIHPNTGFSLLCQQFYAMFL

   901                                                                                               1000
Mm KKITYSRRNWMLVLSVQVLLPLAIIMLSLTFFNFKLRKLDNVPLELTLQTYGQTIVPFFIAENSHLDPQLSDDFVKMLVAAGQVPLRIQGSVEDFLLKKA
Rn KKVAFSRRNWMLVLSVQILLPLVIIMLSLSFFNFKLRKLDNVPLELTLQTYGQTIVPFFIAENSRLDPQLSDNFVKMLVAAGQVPLRIQGSVENFLLKKA
Cf KKITYSWRNWMMLTVQVLVPLAITVFSLAFLNLETR.LDDVPLELTLKTYGQTIVPFYISPNSRLNPQLLEYFTDMLVAEEQIPLETPTLVEDLLLKRA
Bt KRATYSWRNWIMLSIQILVPLVILSISLSFLNFDIS.MDNSPLELTLKSYGQTIVPFYISPNSRLGPRFLDHLTDVLMAEDQKPLELQSPVEAFLLKKL

   1001                                                                                              1100
Mm KEAPEGFDKLYVVAASFEDVNNHTTVKALFNNQAYHSPSLALTLVDNLLFKLLSGANASITTTNYPQPQTAIEVSESILYQGPKGHYLVVNFLFGIAFLS
Rn KEAPEDFDKLYVVAASFEDVNDHTTVKALFNNQAYHSPSLALALVDNVLFKLLSGANASITTTNYPQPQTAMELSETILYQGPKGHYLVVNFLFGIAFLS
Cf EEEPESFDNRYIVAASFEDSGNHTTVTALFNNQAYHSPAVALALVDNVLFKLLSGTRASITAVNHPQPQSAVEALEDILYQGPKGHYLVIHLLFGIAFLS
Bt EEEPEVFDQNYLVAISFDDMENHTVVTALFNNQAYHSTAQALALVDNVLFKLLSGPSASITVLNHPQPESSMETAEDILYEGPKGHYLVINLLFGMAFLS

   1101                                                                                              1200
Mm SSFSILTVGEKSVKSKSLQFVSGVSTAVFWLSALLWDLISFLVPTLLLVLVFLWYKEEAFAHESIPAVVLLMMLYGWAVIPLVYTVSFSFNTPGSACVK
Rn SSFSILTVGEKSIKSKNLQFLSGVSMAAFWLSALLWDLISFLVPTLLLVLVFFWYKEEAFAHPQSIPAVVLIMMLYGWAIIPLVYTVSFSFKTPGSGCVK
Cf SSFSILTVKERATRAKHIQLTSGVYVTTFWLSALLWDLITSLVPSLLLLVVFLHYKEEAFTHKDNIPAVILVLMLYSWASIPFIYLTSFCFQYEGSAFIK
Bt SSFSILTVKERGLKAKQVQFISGVHVATFWLSSLLWDLLSFLVPSLLLLVVFLYYDEEAFTHKENVRAVFLMLMLYAWAIIPFIYLTSFCFENAGNACVK
```

```
      1201                                                                                              1300
Mm    LVVMLTFLSISPVVLVTVTSEKDLGYTELSDSLDHIFLILPGHCLGMALSNLYYNFELKKFCSAKNLSDIDCNDVLEGYVVQENIYAWESLGIGKYLTAL
Rn    LVAMLTFLSISPVVLVTVTSEIDLGYTELSDTLDHIFLIFPGHCLGMAFSNLYYNFEIKKFCNAKNLSDIDCNDVLEGYVVQKNIYAWESLGIGKYLTAL
Cf    LIIILTFLSIGPFILVSVTSEKELGYTTVSDSLDHTFLLLPGHCLGMALFNLYYNHGMQKLCKIKNLSQYECNEISEGFTVQESIYAWESLGMGKYLTAL
Bt    LIIMLTFLSIGPFVLVSVTSEKDLGYTEISESLDNTFLLLPGHCLGMALANLYYNFELQKFCKVKSLDQIECSKVSEGYVVQEDIYAWESLGMGKYLIAL

      1301                                                                                              1400
Mm    AVLGPVYITMLFLTEANAFYVLKSRLSGFFPSFWKEKSGMIFDVAE.PEDEDVLEETEAIKHYLETLIKKNPLVVKEVSKVYKDEVPLLAVNKVSFVVKE
Rn    AILGPVYITLLFLTEANAFCALKARLSGFF...CKQKLRMLLNVTG.AEDEDVLEEAENIKYHLDTLIKKSPLVVKELSKVYKEKVPLLAVNKVSFVVKE
Cf    AILGSVYLIFLFLIETNILRELKARFSTCN...KKRELVTSPDAPSKPLEQDVGQEATTVATYLKKLRQENPLVLHEVSKVYATKVPLLAVNKVSFTVQA
Bt    AISGLVYLILLFLIETNVLWELKARFSGLY...WKQKLVVLQNAESVPGDQDVEEEAKMIKNSWEDLCKKNPLVLKELSKVYEQRAPLLAVDKVSLAVQK

      1401                                                                                              1500
Mm    GECFGLLGLNGAGKTSIFNMLTSEQPITSGDAFVKGFNIKSDIAKVRQWIGYCPEFDALLNFMTGREMLVMYARIRGIPECHIKACVDLILENLLMCVCA
Rn    KECFGLLGLNGAGKTSIFNMLTREQPITSGDAFVKGFNIRTDMAKVQQWIGYCPEFDALLNFMTGREMLVMHARIRGIPECHIKTCVDMILENLLMCVYA
Cf    GECFGLLGLNGAGKTSIFKMLTGDEPITSGDAFLRGLSIRSHIREVRQRIGYCPQFDALLDHMTGRETLVMYARLRGIPERHIAACVENTLRGLLLEPHA
Bt    GECFGLLGFNGAGKTTTFKMLTGEETITSGDAFVGGYSISSDIGKVRQWIGYCPQVDALLDHMTGKETLVMFSRLRGIPERHISSCVDQILDDLLMYTYA
                Walker A
      1501                                                                                              1600
Mm    DKLVKTYSGGNKRMLSTGIALVGEPAVILLDEPSTGMDPVARRLLWDTVERVRESGKTIVITSHSMEECEALCTRLAIMVQGQFECLGSPQHLKSKFGIG
Rn    DKLVKTYSDGNKRVLSTAIALLGEPTVILLDEPSTGMDPVARRLVWDAVGRVRESGKTIVITSHSMEECEALCTRLAIMVQGQFKCLGSPQHLKSRFGSG
Cf    NKLVRTYSGGNKRKLSTGIALLGEPSVIFLDEPSTGMDPMARRLLWSIISRARESGKAIVITSHSMEECEALCTRLAIMVEGQFKCLGSPQHLKSKFGSG
Bt    DKLVKTYSGGNRRKLSAGIALLGEPVVIFLDEPSTGMDPVARRLLWGTVARARKSGKAIVITSHSMEECEALCTRLAIMVQGQFKCLGSPQHLKSKFGSG
                  Signature                Walker B
      1601                                                                                              1700
Mm    YSLQAKVRRKWQQQMLEEFKAFVDLTFPGSNLEDEHQNMLQYYLPGPNLSWAKVFSIMEQAKKDYMLEDYSISQLSLEDIFLNFTRPESSTKEQIQQEQA
Rn    YSLQAKVRRKWQQQMLEEFKAFVDLTFPGSSLEDEHQSMVQYYLPGQNLSWAKVFGIMEQAKKDYVLEDYSISQLSLEDIFLSFTRPVPDTKENIQQGQA
Cf    YSLRAKIRSDKQQEALEEFKAFVNLTFPGSVLEDEHQGMVHYHLPGADLSWARCLAFWSKPRRSSCWTTTLTKSPWRTFSASPALCLSQ.RRKQAQASRG
Bt    YSLRAKIRSDGQQEALEEFKAFVGLTFPGSVLEDEHQGMVHYHLPGDELSWAKVFGIMEQAKTMYMLEDYSVNQISLEDIFLSFSCPVP.PAKGVTDKGG

      1701                                                                       1777
Mm    ALASPSPPSNSRPISSPPS.................RLSSPTPKP....................LPSPPPSEPILL
Rn    ALDSSLSPSNSRPISSPPSSPPSSPPSSPPSRPPSRPSQPPSRPPSRHPSSPSQPPSRPPSRHPSSPSQPPSEPVLL
Cf    FIN..LTPSLFLPTSLPAS.................LSTCLPAS.................LPASLSTCLSLP~~~
Bt    PSP..QTPRFPVHLSLPLP.................PSYPLHLP.................LHPPLHLPPS~~~~~
```

Blue and red shaded amino acids highlight sequence identities. Walker A and B motifs and signature sequences are shaded black. The amino acid sequences of mouse and rat Abca17 were obtained from published GenBank entries (mouse: NP_001026792; rat: NP_001026807). The putative Abca17 polypeptide sequences of the dog and the cow, respectively, were generated on the basis of nucleotide sequence homology searches in the respective genomes using the UCSC Genome Browser. Mm, Mus musculus; Rn, Rattus norvegicus; Cn, Canis familiaris; Bt, Bos taurus.
